# Rosmarinic Acid from Eelgrass Shows Nematicidal and Antibacterial Activities against Pine Wood Nematode and Its Carrying Bacteria

**DOI:** 10.3390/md10122729

**Published:** 2012-11-30

**Authors:** Jingyu Wang, Xueru Pan, Yi Han, Daosen Guo, Qunqun Guo, Ronggui Li

**Affiliations:** Department of Biology, Qingdao University, Qingdao 266071, China; Email: jingyu501@163.com (J.W.); panxueru@sohu.com (X.P.); karissahy@163.com (Y.H.); guodaosen@sohu.com (D.G.); gqunqun@163.com (Q.G.)

**Keywords:** *Zostera marina*, *Bursaphelenchus xylophilus*, rosmarinic acid, nematicidal activity, anti-bacterial activity

## Abstract

Pine wilt disease (PWD), a destructive disease for pine trees, is caused by the pine wood nematode (PWN), *Bursaphelenchus xylophilus* and additional bacteria. In this study, extracts of *Zostera marina* showed a high nematicidal activity against PWN and some of the bacteria that it carries. Light yellow crystals were obtained from extracts of *Z. marina* through solvent extraction, followed by chromatography on AB-8 resin and crystallization. The NMR and HPLC analysis showed that the isolated compound was rosmarinic acid (RosA). RosA showed effective nematicidal activity, of which the LC_50_ (50% lethal concentration) to PWN at 24 h, 48 h and 72 h was 1.18 mg/g, 1.05 mg/g and 0.95 mg/g, respectively. To get a high yield rate of RosA from *Z. marina*, single factor experiments and an L_9_ (3^4^) orthogonal experiment were performed. This extraction process involved 70% ethanol for 3 h at 40 °C. The extraction dosage was 1:50 (w/v). The highest yield of RosA from *Zostera* was 3.13 mg/g DW (dried weight). The crude extracts of *Zostera marina* (10 mg/mL) and RosA (1 mg/mL) also showed inhibitory effects to some bacterial strains carried by PWN: *Klebsiella *sp., *Stenotrophomonas maltophilia*, *Streptomyces *sp. and *Pantoea agglomerans*. The results of these studies provide clues for preparing pesticide to control PWD from *Z. marina*.

## 1. Introduction

Pine wilt disease (PWD) is a global threat to forest resources that is caused by the pine wood nematode (PWN), *Bursaphelenchus xylophilus*, and the bacteria it carries [1,2]. Traditionally, chemicals were used to control this disease. However, since these chemicals have harmful environmental effects, there have been ongoing efforts to use bio-controls derived from natural products. To date, no such products are effective against PWD [3]. Therefore, finding an environmentally friendly pesticide that is effective is critical to control this forest disease. Some natural products from microorganisms and plants have been found to contain nematicidal activity. Effective nematicidal activity against *B. xylophilus* was isolated from essential oils of coriander (*Coriandrum sativum*), oriental sweetgum (*Liquidambar orientalis*) and valerian (*Valeriana wallichii*) [4]. Several compounds with nematicidal activity have been isolated and identified from ajowan (*Trachyspermum ammi*), allspice (*Pimenta dioica*) and litsea (*Litsea cubeba*) [5]. Ingenane diterpenes from *Euphorbia kansui* and their derivatives also showed good nematicidal activity against the pine wood nematode [6]. Zhang *et al.* [7] found that the ethanol extract of *Liriope muscari* fibrous roots possessed significant nematicidal activity against the pine wood nematode. The compound (1,4-epoxy-*cis*-eudesm-6-*O*-β-D-glucopyranoside) and two known glycosides with nematicidal activity had been isolated from ethanol extracts. Guo *et al.* [8] found that the ethanol extract of fennel could kill PWN and its associated bacteria.

Eelgrass (*Zostera marina*) is a flowering angiosperm belonging to Potamogetonaceae, which is widely distributed in coastal marine habitats in the northern hemisphere. Since intact eelgrass leaves decay very slowly [9], they are widely selected and used as roof materials in some seaside villages in Northern China. In Russia, eelgrass is widely used as food and drugs [10]. Previous studies indicated that water-soluble extracts of eelgrass leaves could inhibit growth of micro-algae, marine bacteria and grazing by amphipods on dead leaves [11,12]. These observations indicate that eelgrass is a good resource for screening natural antibiotics. In this study, an effective compound with nematicidal and antibacterial activity was isolated from eelgrass extracts, and its effectiveness was investigated.

## 2. Results and Discussion

### 2.1. Nematicidal Activity of Eelgrass Extracts

Different fractions of eelgrass extracts were evaporated, weighed and resuspended in water at various concentrations. These crude extracts showed different nematicidal activities against PWN when used at various concentrations (Table 1). Crude extracts from the ethyl acetate fraction exhibited high nematicidal activity, particularly when used at 25 mg/mL, with a death rate of 100%. The chloroform fraction also showed some toxicity to nematodes, with a 65.4% death rate when used at 30 mg/mL. 

### 2.2. Elucidation the Structure of Rosmarinic Acid

**Figure 1:**
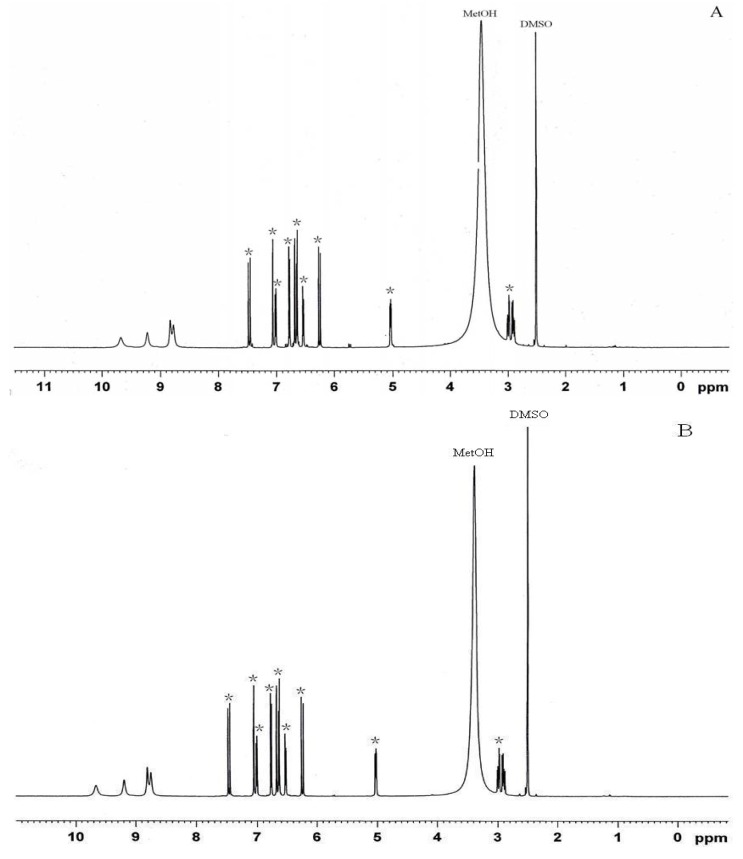
^1^H-NMR spectra (DMSO, 500 MHz) of (**A**) rosmarinic acid from *Z. marina* and (**B**) rosmarinic acid standard. * stands for rosmarinic acid signals.

To isolate the compounds that possessed nematicidal activity, the ethyl acetate fraction of the eelgrass extract was further separated by chromatography on an AB-8 column. Light yellow crystals were obtained after crystallization. The ^1^H-NMR data for this compound and rosmarinic acid standard were shown in Figure 1.

**Table 1:** Nematicidal activities of crude *Z. marina* extracts. ^a–d^ Values stand for the corrected death rate (%) of nematode, which are expressed in mean ± standard deviation(SD) of 4 parallels. Means with the same letters are not significantly different at *P* < 0.05.

Concentration (mg/mL)	Yield rate (%)	5	10	15	20	25	30
Petroleum ether fraction	4.6	2.3 ± 0.12 ^d^	2.8 ± 0.14 ^c^	3.3 ± 0.55 ^c^	9.9 ± 0.43 ^d^	20.3 ± 0.43 ^d^	26.7 ± 1.10 ^d^
*n*-Butanol fraction	15.2	6.7 ± 0.44 ^c^	7.1 ± 0.02 ^b^	15.3 ± 1.98 ^b^	19.6 ± 0.27 ^c^	33.2 ± 0.98 ^c^	46.7 ± 1.46 ^c^
Chloroform fraction	7.4	7.3 ± 0.21 ^b^	7.7 ± 1.00 ^b^	13.5 ± 1.54 ^b^	29.2 ± 0.49 ^b^	47.6 ± 1.19 ^b^	65.4 ± 2.57 ^b^
Ethyl acetate fraction	21.3	12.0 ± 0.43 ^a^	23.7 ± 0.97 ^a^	57.4 ± 1.12 ^a^	87.6 ± 0.55 ^a^	100 ± 0.00 ^a^	100 ± 0.00 ^a^

**Figure 2:**
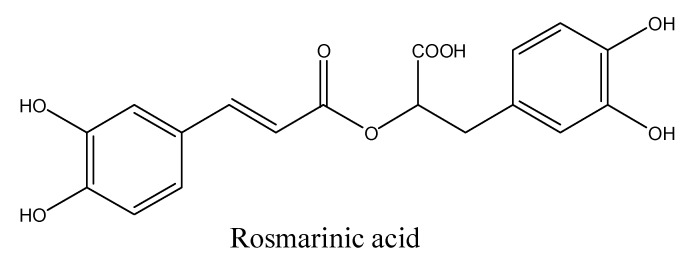
Chemical structure of rosmarinic acid.

Based on the data collected and comparing to other published spectral values, the purified compound was identified as rosmarinic acid (RosA). The chemical formula for this compound is shown in Figure 2.

**Figure 3:**
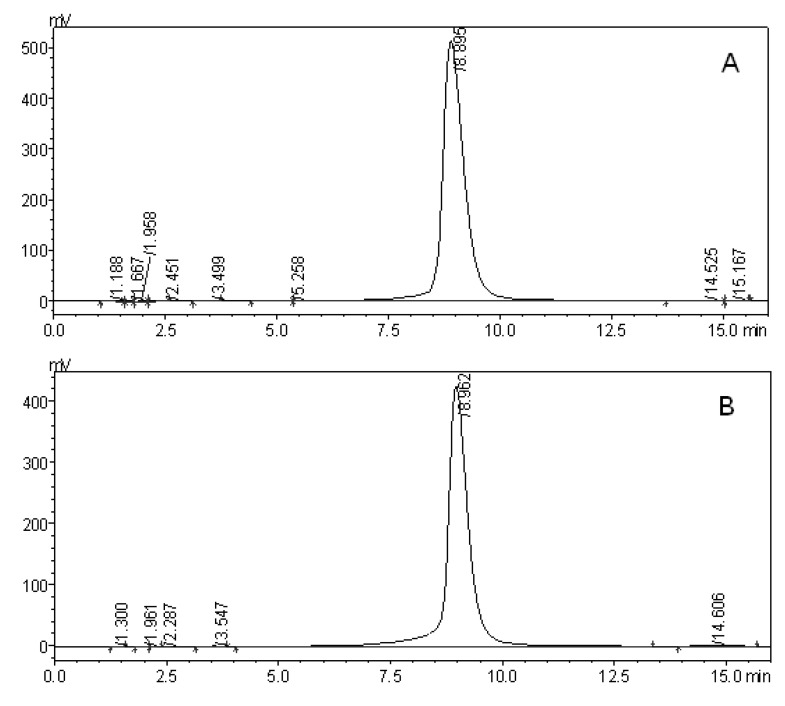
HPLC analysis of (**A**) rosmarinic acid from *Z. marina* and (**B**) rosmarinic acid standard. The samples were passed through reversed phase column VP-ODS C18 (150 × 4 mm) at 1 mL/min. Chromatograms were monitored at 330 nm, and the mobile phase was methanol/water/acetic acid (55:44.9:0.1, v:v:v). The experiment was carried out at 30 °C. The retention time of rosmarinic acid was approximately 8.9 min.

HPLC analysis showed that the isolated crystal component had similar retention times to the RosA standard (Figure 3). This observation provided further validation that the extracted compound from eelgrass is RosA, with a purity of 97.9% as determined by HPLC.

### 2.3. Nematicidal Activity of RosA

RosA isolated from *Z. marina *showed effective nematicidal activity *in vitro*. The LC_50_ (50% lethal concentration) of RosA is 1.18, 1.05 and 0.95 mg/mL at 24, 48 and 72 h, respectively (Table 2). There was not much difference of RosA at LC_50_ at 24 h, 48 h and 72 h, indicating RosA has rapid nematicidal activity. Compared with RosA, copper sulfate had stronger nematicidal activity (Table 2).

Table 2Nematicidal activity of RosA. **A**—Nematicidal activity of RosA; **B**—Result analysis of RosA nematicidal activity. ^a–f^ Values stand for the corrected death rate (%) of nematode, which are expressed in mean ± standard deviation (SD) of 4 parallels. Means with the same letters are not significantly different at *P* < 0.05. The formula *y *= *bx *+ *a* stands for the linear regression curve. CL stands for confidence limit.

**Table:** A

	Concentration (mg/mL)	24 h	48 h	72 h
Rosmarinic acid	0.5	29.83 ± 1.38 ^f^	33.41 ± 4.55 ^f^	37.81 ± 1.21 ^e^
	1	43.23 ± 2.20 ^e^	50.26 ± 3.02 ^e^	54.65 ± 2.90 ^d^
	1.5	65.80 ± 0.85 ^d^	69.63 ± 2.02 ^d^	74.31 ± 2.02 ^c^
	2	73.25 ± 4.82 ^c^	84.66 ± 0.98 ^c^	89.35 ± 3.24 ^b^
	2.5	79.91 ± 3.89 ^b^	94.30 ± 0.65 ^b^	98.29 ± 0.87 ^a^
	3	86.84 ± 2.69 ^a^	99.40 ± 1.26 ^a^	100.00 ± 0.00 ^a^
Copper sulfate	0.05	27.45 ± 0.31 ^f^	39.45 ± 0.39 ^f^	44.05 ± 0.86 ^e^
	0.1	37.49 ± 0.02 ^e^	51.08 ± 2.20 ^e^	54.87 ± 2.20 ^d^
	0.15	44.70 ± 1.26 ^e^	58.78 ± 1.03 ^e^	78.04 ± 0.98 ^c^
	0.2	54.74 ± 0.92 ^d^	84.05 ± 0.87 ^c^	90.41 ± 1.02 ^b^
	0.25	78.88 ± 1.12 ^b^	99.91 ± 0.02 ^a^	100.00 ± 0.00 ^a^
	0.3	91.12 ± 1.00 ^a^	100.00 ± 0.00 ^a^	100.00 ± 0.00 ^a^

**Table:** B

	*t* (h)	*y* = *bx* + *a*	*r*	LC_50_ (mg/mL)	CL of LC_50_
Rosmarinic acid	24	*y* = 0.230*x* + 0.229	0.971	1.18	(1.09, 1.27)
	48	*y* = 0.251*x* + 0.236	0.954	1.05	(0.97, 1.13)
	72	*y* = 0.254*x* + 0.258	0.946	0.95	(0.90, 1.00)
Copper sulfate	24	*y* = 2.524*x* + 0.112	0.982	0.154	(0.144, 0.164)
	48	*y* = 3.081*x* + 0.205	0.981	0.096	(0.090, 0.102)
	72	*y* = 3.245*x* + 0.263	0.989	0.073	(0.069, 0.077)

### 2.4. Optimization of RosA Extraction Conditions from Eelgrass

**Figure 4:**
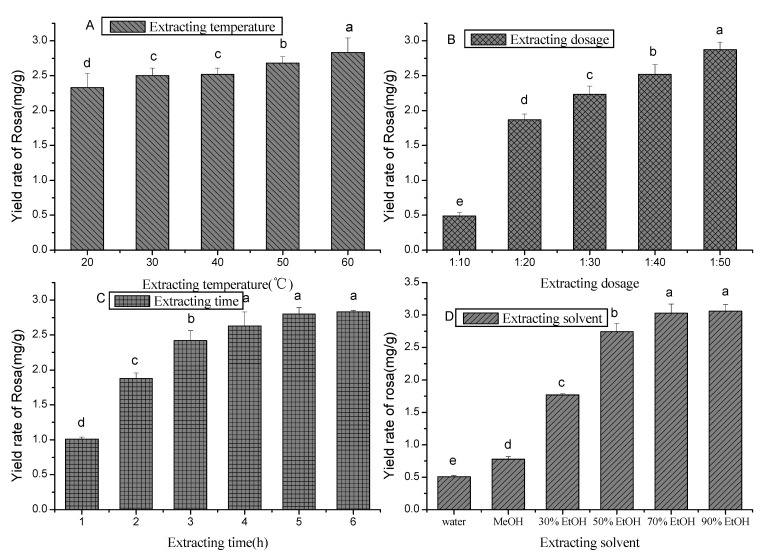
Single factor experiment result. **A**—extracting temperature, **B**—extracting dosage, **C**—extracting time, **D**—extracting solvent. ^a–e^ Means with the same letters are not significantly different at *P* < 0.05.

To extract RosA efficiently from *Z. marina*, we optimized the extraction solvent, temperature, time and solid-liquid ratio (Figure 4). Compared to water and methanol, ethanol yielded a higher rate of RosA at different concentrations. A 70% ethanol concentration yielded at least 3.03 mg/g of RosA, while further increases in ethanol concentration did not raise the yield rate. Temperature increase also yielded a higher rate of RosA from 2.33 mg/g at 20 °C to 2.83 mg/g at 60 °C. Prolonged extraction time also increased RosA concentration, with the yield rate being stable when the extraction time reached 5 h. The yield rate of RosA also increased when decreasing the solid-liquid ratio from 0.49 mg/g (1:10, w/v) to 2.87 mg/g (1:50, w/v).

The L_9_ (3^4^) orthogonal experiment was performed based on each parameter described above that affects extraction efficacy. The optimal conditions to increase RosA extraction is by using an extraction dosage at 1:50 and using 70% ethanol with an extraction time of 3 h at 40 °C (Table 3). A validation experiment was performed using these optimized conditions, and RosA was extracted at a concentration of 3.13 mg/g. 

### 2.5. The Antibacterial Activity of Crude Eelgrass Extracts and RosA

The antibacterial activity of eelgrass extracts and RosA was also tested on bacteria associated with PWN. The petroleum ether, ethyl acetate and chloroform fractions were able to differentially inhibit the growth of the four bacteria commonly found in PWN. Comparatively, the *n*-butanol fraction showed no obvious inhibition to *Pantoea agglomerans* and *Stenotrophomonas maltophilia* and could only slightly inhibit *Klebsiella *sp. and *Streptomyces *sp. RosA was found most effective against all four bacteria when used at a low concentration (1 mg/mL), but less effective than oxolinic acid (Table 4).

Table 3The L_9_ (3^4^) orthogonal experiment for the extraction of RosA. **A**—The orthogonal experiment result, **B**—Variance analysis result of the L_9_ (3^4^) orthogonal experiment.

**Table:** A

Experiment No.	Dosage	Time	Temperature	Solvent	Yield rate
	(w:v)	(h)	(°C)	(%)	(mg/g)
1	1(1:30)	1(3)	1(30)	1(30)	2.29
2	1(1:30)	2(4)	2(40)	2(50)	2.53
3	1(1:30)	3(5)	3(50)	3(70)	2.80
4	2(1:40)	1(3)	2(40)	3(70)	2.89
5	2(1:40)	2(4)	3(50)	1(30)	2.35
6	2(1:40)	3(5)	1(30)	2(50)	2.64
7	3(1:50)	1(3)	3(50)	2(50)	2.72
8	3(1:50)	2(4)	1(30)	3(70)	2.78
9	3(1:50)	3(5)	2(40)	1(30)	2.46
M1	M11 = 2.540	M12 = 2.633	M13 = 2.570	M14 = 2.367	
M2	M21 = 2.627	M22 = 2.553	M23 = 2.627	M24 = 2.630	
M3	M31 = 2.653	M32 = 2.633	M33 = 2.623	M34 = 2.823

**Table:** B

Factors	S	F	F ratio
Dosage	S1 = 0.021	2	0.434
Time	S2 = 0.013	2	0.047
Temperature	S3 = 0.006	2	0.124
Solvent	S4 = 0.315	2	3.395
error	0.350	2	

**Table 4:** Antibacterial activity of four eelgrass extracts and RosA. The value stands for the whole diameter of the inhibitory rings, including the 6 mm filter paper. The value 0 means no inhibitory rings exist on the medium.

Bacteria	Diameter of inhibitory rings (mm)					
	Petroleum ether fraction (10 mg/mL)	*n*-Butanol fraction (10 mg/mL)	Chloroform fraction (10 mg/mL)	Ethyl acetate fraction (10 mg/mL)	RosA (1 mg/mL)	Oxolinic acid (0.1 mg/mL)
*Klebsiella *sp.	26	12	22	25	28	12
*Stenotrophomonas maltophilia*	13	0	12	16	19	10
*Streptomyces *sp.	16	8	20	30	26	10
*Pantoea agglomerans*	14	0	19	18	18	19

*Z. marina* is an aquatic plant found in wide grasslands that contributes to the coastal ecosystems. It does this by generating substantial amounts of organic matter, while providing a habitat for many organisms [13]. However, the residues reaching the coastlines pose an environmental problem with high costs for their disposal. Eelgrass residue was investigated for bioethanol, and 8.72% bioethanol could be produced using an integrated process [14]. Zosterin, bioactive pectin from the eelgrass *Z. asiatica* decreases the toxicity of antitumor drugs and purges heavy metals from human organisms [15,16]. Kawasaki *et al. *[17] analyzed compounds in the essential oil from eelgrass shoots by GC and GC mass spectrometry and found that the major constituents were phytol, hexadecanamide, octadecanamide, pentadecane, heptadecane, nonadecane, (8*Z*,11*Z*)-heptadecadienal, (8*Z*)-heptadecenal, (9*Z*,12*Z*,15*Z*)-octadecatrienal and (9*Z*,12*Z*)-octadecadienal. Achamlale *et al.* [18] isolated and quantified RosA in *Z. noltii* and *Z. marina* by high performance liquid chromatography, and found concentrations of RosA ranging from 2.2 to 18.0 mg/g (dw) for *Z. noltii* and 1.3 to 11.2 mg/g (dw) for *Z. marina*. These results show great value in using *Zostera* for extracting compounds with anti-bacterial and nemacitidal activities.

RosA is a multifunctional polyphenol that was first isolated from the leaves of *Rosmarinus officinalis* and later found in other species of Lamiaceae and Boraginaceae. RosA has been demonstrated to have higher antioxidant activity that is more effective than Vitamin E [19,20]. Other biological activities, such as antiviral [21], antibacterial [22,23], anti-allergenic [24] and anticarcinogenic effects within the body [25] have also been reported. The use of RosA has ramifications to benefit different field services, including the food, pharmacy and cosmetics industries. In this study, we found that the ethanol extract from eelgrass and RosA showed effective nematicidal activity against PWN and antibacterial activity to four bacteria associated to PWN. This is the first report of nematicidal activity extracted from eelgrass and RosA. Overall, eelgrass may serve as a new resource for screening compounds that are structurally similar to RosA, as well as additional compounds with nematicidal activity.

## 3. Experimental Section

### 3.1. Preparation of Eelgrass, Nematode and Bacteria

Live eelgrass (*Z. marina*) was collected in June to August in 2011 at low tide from the sub-tidal beds at Qingdao, China (36.10°N, 120.40°W) .The eelgrass leaves were washed and dried naturally at room temperature and then crushed in smaller pieces by a disintegrator.

Pine wood nematodes were isolated from a piece of wood cut from a pine wilt diseased *Pinus thunbergii*. The nematodes were cultured on the fungus, *Botrytis cinerea*, as described by Zhao *et al.* [1]. The PWN suspension used in the experiment was prepared by washing the plates with sterile water.

Four bacteria carried by PWN, namely *Klebsiella *sp., *S. maltophilia*, *Streptomyces *sp. and *P. agglomerans*, were isolated and identified as described by Guo *et al.* [8].

### 3.2. Preparation of Extraction of *Z. marina*

To prepare an eelgrass extract, 100 g eelgrass homogenate was mixed with 3000 mL anhydrous ethanol, extracted for 24 h at room temperature and filtrated with filter paper. After the ethanol in the filtrate was evaporated, the remaining residue was fully suspended in 300 mL distilled water and extracted three times by petroleum ether, *n*-butanol, ethyl acetate and chloroform, respectively. Each fraction was also evaporated to dryness with a rotary evaporator and saved for further research. The yield rate of each fraction was calculated.

### 3.3. Nematicidal Activity of Crude Eelgrass Extracts

Each fraction was weighed and resuspended in distilled water to a concentration of 5, 10, 15, 20, 25 and 30 mg/mL, respectively. To check the nematicidal activity of each fraction, 20 µL nematode suspension was added to 1 mL solution in a 1.5 mL eppendorf tube. The tube was then cultured at 28 °C in the dark for a period of time. Each experiment was performed in quadruplicate. The numbers of live and killed nematodes were counted by a stereomicroscope, and the corrected death rate of nematode at 24 h, 48 h and 72 h was calculated according to the following formula:

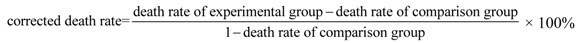
(1)


The results were analyzed by SPSS 17.0. 

### 3.4. Isolation and Identification of Nematicidal Component

The most active component in the ethyl acetate fraction was further purified by AB-8 macroporous adsorptive resin and crystallization. The ethyl acetate fraction suspended in water was passed through a AB-8 resin column (2.5 × 20 cm) (Nankai University Chemical Co. Ltd.) and then eluted by ethanol with a concentration of 10%, 30%, 50% and 70%, respectively. Each eluted fraction was concentrated by evaporating the ethanol and stored at 4 °C for crystallization. Light yellow crystals were obtained from 30% ethanol fraction, and its structure was elucidated by NMR on a Bruker AV-400. ^1^H-NMR identified that the compound as rosmarinic acid [12].

### 3.5. HPLC Analysis

The light yellow crystal was dissolved in ethanol and analyzed by high-performance liquid chromatography on a VP-ODS C18 column (150 × 4 nm, Shimadzu) at 1 mL/min. The detecting wave length was 330 nm, and the moving phase was methanol/water/glacial acetic acid (55:44.9:0.1, v:v:v). RosA (Sigma) was used as the standard.

### 3.6. Nematicidal Activity of RosA

RosA was dissolved in water to a concentration of 0.5, 1, 1.5, 2, 2.5 and 3 mg/mL. Twenty micro liters of pine wood nematode suspension was added to 1 mL RosA solution at final concentrations of 0.5, 1, 1.5, 2, 2.5 and 3 mg/mL, respectively. The final number of nematode was about 10,000 w/mL. Each experiment was performed at least four times. After culturing at 28 °C, the number of live and killed nematodes was counted, and the corrected death rate of nematodes at 24 h, 48 h and 72 h was calculated according to the formula described above. Copper sulfate was used as positive control. The results were analyzed by SPSS 17.0.

### 3.7. Factors Influencing the Extraction of RosA

To obtain high yield rates of RosA from eelgrass, factors influencing extraction of RosA were studied. Extraction temperature, extraction solvent, extraction time and extraction dosage were investigated. RosA content in each extract was measured using FeSO_4_ colorimetric method as described previously [26]. Every experiment was replicated trice. Based on the results of single factor experiment, an L_9_ (3^4^) orthogonal experiment was also performed. 

### 3.8. Inhibition Effects of Eelgrass Extracts and RosA on Four Bacterial Strains Carried by PWN

Four dried fractions were suspended in water to a final concentration of 10 mg/mL, and RosA was dissolved in water to prepare a solution of 1 mg/mL. Filter paper with a diameter of 6mm was immersed in each solution and then put on the surface of nutrient agar medium, which had been inoculated with one of the bacterial strains (*Klebsiella *sp., *S. maltophilia*, *Streptomyces *sp. and *P. agglomerans*). After culturing at 37 °C for 24 h, the diameters of the inhibitory rings were measured, and oxolinic acid was used as positive control. 

## 4. Conclusions

In summary, extracts of *Z. marina *showed good nematicidal activity and anti-bacterial activity against pine wood nematode and its associated four bacterial strains. An active compound was isolated from the eelgrass extracts and identified as rosmarinic acid. To prepare this compound efficiently, the extraction process of rosmarinic acid from *Z. marina* was optimized, and the highest yield was 3.13 mg/g DW under the ideal extract conditions. 
